# Keratouveitis as a First Presentation of Relapsing Polychondritis

**DOI:** 10.1155/2010/176514

**Published:** 2010-10-28

**Authors:** Ahmed Sallam, Tahmina Islam, Dipak N. Parmar

**Affiliations:** Department of Ophthalmology, Whipps Cross University Hospital, London E11 1NR, UK

## Abstract

This paper provides images and a description of an unusual manifestation of relapsing polychondritis presenting initially with isolated ocular signs, mimicking infective keratitis. We present an interventional case report of a 75-year-old man who presented with marked left ocular irritation and photophobia. Ophthalmological examination disclosed corneal intrastromal infiltrate and hypopyon which failed to respond to intensive antimicrobial drops. He later went on to develop bilateral auricular chondritis. Relapsing polychondritis was diagnosed. Treatment with topical and oral corticosteroids resulted in marked improvement of the corneal infiltrate and resolution of the auricular inflammation. The paper highlights the importance of considering connective tissue inflammatory conditions in any stromal keratitis unresponsive to antimicrobial treatment.

## 1. Case Report

**Figure 1:**
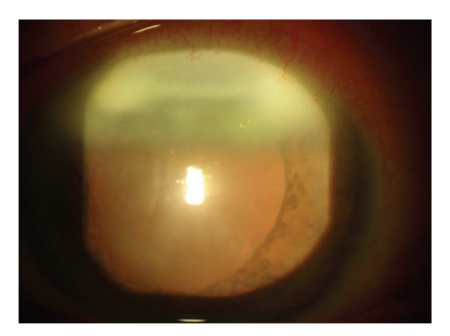
Slit lamp photography showing dense superior intrastromal corneal infiltrate.

A 75-year-old Caucasian man presented with marked irritation and photophobia in his left eye of 3-week duration. Snellen acuity at best was 6/24 OS and 6/12 OD. Biomicroscopic examination revealed a 3 mm superior corneal intrastromal infiltrate, with no associated overlying epithelial defect. There was intense anterior chamber activity with a 1 mm hypopyon (Figure 1). A corneal scrape was taken at the time but failed to show any growth on standard culture media.

Despite hourly ofloxacin therapy for 24 hours, the corneal stromal infiltrate remained unchanged and visual acuity then deteriorated to 6/60 in the left eye. He complained of auricular pain, redness, and swelling of both earpinnae, but the ear lobules were spared. Erythrocyte Sedimentation Rate (ESR), C-reactive protein (CRP), full blood count, renal and liver function tests, and autoantibody screen all proved normal.

**Figure 2:**
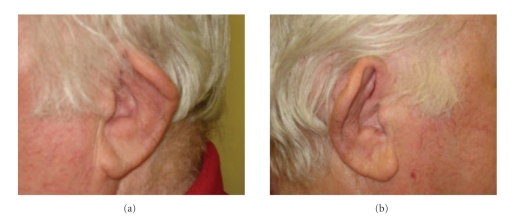
Deformed ear pinnae following the resolution of auricular chondritis.

 A marked clinical response was observed with topical hourly prednisolone forte and oral prednisolone 40 mg daily. Within two days of treatment, resolution of the hypopyon was noted alongside reduction of cutaneous vasculitis and auricular chondritis (Figure 2). By week 3, the left cornea was clear and visual acuity had improved to 6/6. Systemic steroid was gradually tapered to a maintenance dose of 5 mg per day by 2 months, with no further signs of active disease.

## 2. Discussion

Relapsing polychondritis is a rare autoimmune connective tissue disorder affecting primarily cartilaginous tissues [1]. These include large vessels such as the aorta, respiratory passages, and joints [2]. Although usually indolent with multiple exacerbations, it may become rapidly fatal especially if inadequately treated or diagnosed [2, 3]. 

The most common early signs of RPC are auricular chondritis, and polyarthritis occurring in over 80% of patients [1]. Common ocular manifestations include episcleritis or scleritis, uveitis, keratitis and keratoconjunctivitis sicca [3, 4]. Ocular signs usually occur in conjunction with systemic disease and have been correlated with disease activity [5]. This case is an unusual initial manifestation of RPC, presenting initially with isolated ocular signs. It highlights the importance of considering connective tissue inflammatory conditions in any stromal keratitis unresponsive to topical antimicrobial therapy. A systemic enquiry is invaluable in avoiding a delayed diagnosis and the subsequent associated mortality [3].
